# Metoprolol Reduces Proinflammatory Cytokines and Atherosclerosis in ApoE^−/−^ Mice

**DOI:** 10.1155/2014/548783

**Published:** 2014-07-08

**Authors:** Marcus A. Ulleryd, Evelina Bernberg, Li Jin Yang, Göran M. L. Bergström, Maria E. Johansson

**Affiliations:** ^1^Department of Physiology, Institute of Neuroscience and Physiology, Sahlgrenska Academy, University of Gothenburg, P.O. Box 432, 405 30 Gothenburg, Sweden; ^2^The Wallenberg Laboratory, Department of Molecular and Clinical Medicine/Clinical Physiology, Institute of Medicine, Sahlgrenska Academy, University of Gothenburg, Sahlgrenska University Hospital, 413 45 Gothenburg, Sweden

## Abstract

A few studies in animals and humans suggest that metoprolol (*β*1-selective adrenoceptor antagonist) may have a direct antiatherosclerotic effect. However, the mechanism behind this protective effect has not been established. The aim of the present study was to evaluate the effect of metoprolol on development of atherosclerosis in ApoE^−/−^ mice and investigate its effect on the release of proinflammatory cytokines. Male ApoE^−/−^ mice were treated with metoprolol (2.5 mg/kg/h) or saline for 11 weeks via osmotic minipumps. Atherosclerosis was assessed in thoracic aorta and aortic root. Total cholesterol levels and Th1/Th2 cytokines were analyzed in serum and macrophage content in lesions by immunohistochemistry. Metoprolol significantly reduced atherosclerotic plaque area in thoracic aorta (*P* < 0.05 versus Control). Further, metoprolol reduced serum TNF*α* and the chemokine CXCL1 (*P* < 0.01 versus Control for both) as well as decreasing the macrophage content in the plaques (*P* < 0.01 versus Control). Total cholesterol levels were not affected. In this study we found that a moderate dose of metoprolol significantly reduced atherosclerotic plaque area in thoracic aorta of ApoE^−/−^ mice. Metoprolol also decreased serum levels of proinflammatory cytokines TNF*α* and CXCL1 and macrophage content in the plaques, showing that metoprolol has an anti-inflammatory effect.

## 1. Introduction

Cardiovascular events are most often caused by complications to atherosclerotic disease. *β*-Blockers have been shown to reduce the risk of cardiovascular events after myocardial infarction [1, 2]. The mechanisms behind this cardioprotective effect have been attributed to the many positive effects that *β*-blockers have on cardiac function: antiarrhythmic effects, improvement of myocardial function, lowering of cardiac oxygen consumption, and lowering of blood pressure. In addition, a few studies have also suggested that *β*-blockers may have direct antiatherosclerotic effects in rabbits and monkeys, as well as in humans [3, 4].

In landmark studies, Kaplan and coworkers showed that atherosclerosis development was accelerated in dominant male cynomolgus monkeys living in unstable hierarchies [5, 6]. The effect on atherosclerosis was inhibited by nonselective *β*-blockade using propranolol [7]. In later experiments, blockade with metoprolol protected against acute endothelial injury induced by unstable social conditions in the same animal model, suggesting that stress-induced atherogenesis is mediated via the *β*_1_-receptor [8]. The protective effect of metoprolol was also seen under nonstressed conditions in rabbit models of atherosclerosis [9]. It has also been shown in two separate studies that low-dose metoprolol administered during three years slows the progression of intima media thickness (IMT) in humans [10, 11] and alters the grey scale of carotid plaques [12]. However, despite these convincing data, the mechanisms behind this protective effect have not yet been established.

We hypothesized that metoprolol would reduce progression of atherosclerosis in ApoE^−/−^ mice and therefore studied whether long-term treatment with metoprolol would reduce plaque area. We also studied the potential effects of metoprolol on proinflammatory cytokines known to play a role in development of atherosclerosis.

## 2. Methods

### 2.1. Animals

Mice were housed at 21 to 24°C in a room with a 12 h light/12 h dark cycle. Water and food were available* ad libitum*. All procedures involving mice were approved by the Regional Animal Ethics Committee at the University of Gothenburg, in accordance with the European Communities Council Directives of 24 November 1986 (86/609/ECC).

### 2.2. Study I: Dose-Finding

#### 2.2.1. Experimental Design

To find an appropriate dose of metoprolol we randomly divided male C57BL/6 mice (Taconic, Denmark), 9 weeks of age, into four groups: (i) Control (*C*, *n* = 4), (ii) Metoprolol Dose 1 (1.4 mg/kg per hour, Sigma Aldrich St. Louis, Missouri, USA, *n* = 4), (iii) Metoprolol Dose 2 (2.5 mg/kg per hour, *n* = 4), and (iv) Metoprolol Dose 3 (4.1 mg/kg per hour, *n* = 4).

At 10 weeks of age, mice were implanted with ECG telemetry probes (PhysioTel Transmitters TA-F20, weight 3.9 g, Data Science International, Inc., St. Paul, MN, USA) as previously described [13]. At 12 weeks of age osmotic minipumps (Alzet model 1002, DURECT Corporation, ALZET Osmotic Pumps, Cupertino, CA, USA) with the three different doses of metoprolol were implanted as previously described [14]. Mice were anesthetized with isoflurane for 5–10 minutes and minipumps were implanted subcutaneously on the back of the mouse.

#### 2.2.2. Effects of Metoprolol on Heart Rate

At 13 weeks of age heart rate was measured with radiotelemetry in an undisturbed room during 24 hours at baseline conditions. Further, heart rate was recorded one hour prior to air-jet stress (baseline) and two hours during air-jet stress (stress) for baseline and stress measurements, respectively, as previously described [13].

#### 2.2.3. Plasma Concentration of Metoprolol

Plasma concentrations of metoprolol were measured with liquid chromatography coupled with tandem mass spectrometry (LC/MS/MS) from blood samples collected at termination.

### 2.3. Study II: Effects on Atherosclerosis

#### 2.3.1. Experimental Design

Male ApoE^−/−^ mice (Taconic Transgenic Models Strain B6.129P2-Apoe^tm1Unc^/N11 Taconic, Denmark), 6 weeks of age, were randomly divided into two groups: (i) Control (*C*, *n* = 12) and (ii) Metoprolol infusion (2.5 mg/kg per hour, Met, *n* = 12). From 9 weeks of age and throughout the experiment, mice in both groups were fed a high fat, cholesterol enriched diet, “western diet” (21% fat, 0.15% cholesterol; R638, Lantmännen, Sweden).

At 9 weeks of age, mice were implanted with osmotic minipumps (Alzet model 2006) delivering saline or metoprolol, as described above. Since the duration of the minipumps was six weeks, minipumps were replaced once during the experiment.

#### 2.3.2. Termination and Fixation

The mice in the atherosclerosis study were sacrificed at 19 weeks of age with an overdose of pentobarbital (Apoteksbolaget, Sweden, 0.9 mg/g BW i.p.), as previously described [15]. Briefly, blood was collected from the right ventricle into standard coated tubes for Li-heparin and serum. The heart and vascular tree were perfused by intracardiac saline infusion to clear the lumen from blood. The heart and aorta were then fixed in 4% paraformaldehyde. After fixation, the thoracic aorta (from the left common carotid artery to the right renal artery) was cleared from surrounding fat and tissue and kept in 4% paraformaldehyde.

### 2.4. Quantification of Atherosclerosis and Immunohistochemistry

#### 2.4.1. *En Face* Quantification of Thoracic Aorta

The thoracic aorta was analyzed* en face*, as previously described [15]. Briefly, the aortas were cut open longitudinally, pinned onto silicone dishes, and stained with Sudan IV for lipids. Images were captured with Canon Utilities Remote Capture 2.7 (Canon Inc., Tokyo, Japan) using a digital camera connected to a dissection microscope. The outline of the intima was manually traced using Adobe Photoshop, by a blinded observer, to calculate the total area of the vessel. Lesions were outlined in the same manner and plaque area was calculated as the percentage of the total vessel area covered with lesions.

#### 2.4.2. Cross-Sectional Quantification of Aortic Root

The aortic root was serially sectioned and stained with Oil Red O as previously described [16]. Briefly, the aortic root was serially sectioned at six different levels, 100–600 *μ*m from the aortic valves. The cross-sections of the vessel were then stained with Oil Red O and lesions were measured by a blinded observer. Lesion size was normalized by IEL length.

#### 2.4.3. Immunohistochemistry

Immunostaining was performed on formalin-fixed cross-sections of the aortic root as previously described [14, 17]. The following antibodies were used: Mac-2 (CL8942AP: Cedarlane Laboratories Ltd., Burlington, Ontario, CA) and biotinylated rabbit anti-rat IgG (BA-4001: Vector Laboratories, Burlingame, CA, USA). Binding was visualized by DAB kit (Vector Laboratories) and counterstained with Hematoxylin (HARRIS HTX Histolab: Histolab Procuts AB, Gothenburg, Sweden). Images were produced with an Olympus BX60F5 microscope with a 10X objective connected to an Olympus DP72 camera. Positive staining for Mac-2 was automatically traced using cellSens Dimension analysis software (Version 1.5, Olympus Optical Company, Hamburg, Germany) and normalized to lesion area. Data are presented as the average staining from two consecutive sections.

### 2.5. Blood Sample Analysis

#### 2.5.1. Analysis of Th1 and Th2 Cytokines

Serum samples were used for analysis of Th1 cytokines: IL-1*β*, IL-2, IL-12 total, IFN-*γ*, TNF*α*, and CXCL1 and Th2 cytokines: IL-4, IL-5, and IL-10, using a Mouse Th1/Th2 Multiplex ELISA (Meso Scale Discovery, Gaithersburg, Maryland, USA) according to the manufacturer's protocol.

#### 2.5.2. Total Cholesterol

Total serum cholesterol was determined colorimetrically after enzymatic hydrolysis and oxidation using a cholesterol kit (cholesterol enzymatic endpoint method, RANDOX Laboratories Ltd., United Kingdom), according to the manufacturers protocol.

### 2.6. Statistics

24-hour heart rate was analyzed using repeated measurement ANOVA followed by Dunnett's post hoc test (SPSS Statistics version 17.0, Chicago, IL, USA). All other data was analyzed with the nonparametric Mann-Whitney *U* test (SPSS). One mouse in the metoprolol treated group in Study II was considered an outlier in SPSS regarding plaque area. This mouse was removed from all analysis in the study. All data are expressed as mean ± SEM. *P* < 0.05 was considered as statistically significant for all data except for Th1/Th2 cytokines. To reduce the risk for mass significance in the analysis of Th1/Th2 cytokines we used a 99% significance level (*P* < 0.01) for this data.

## 3. Results

### 3.1. Study I: Dose-Finding

#### 3.1.1. Metoprolol Lowered 24-Hour Heart Rate

**Figure 1:**
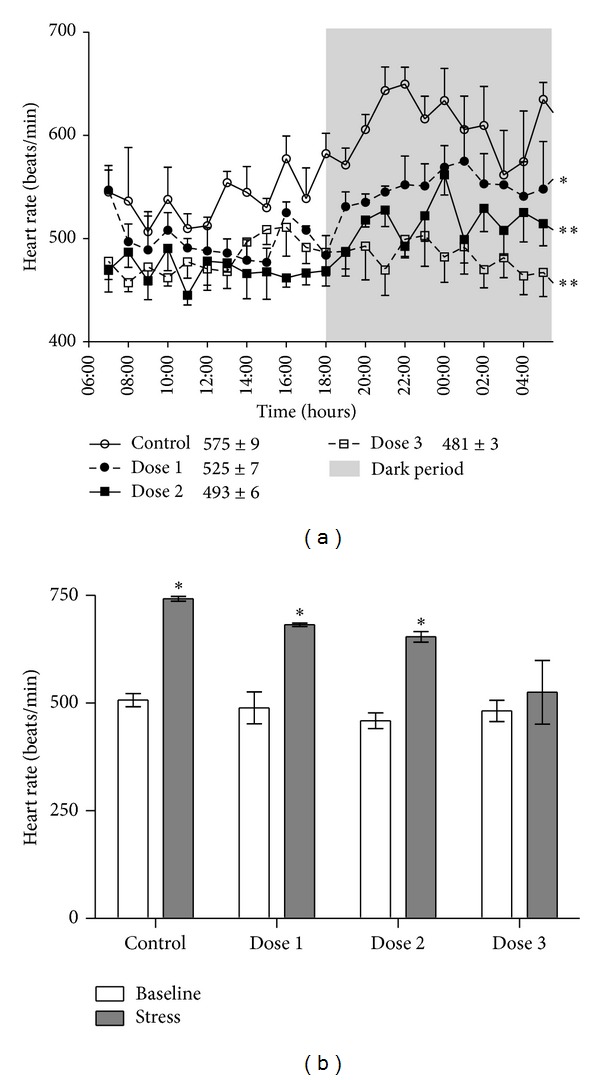
Metoprolol dose-finding (Study I). (a) 24-hour heart rate during baseline conditions after three different doses of metoprolol compared with Control mice. Metoprolol was administrated to C57BL76 mice via osmotic minipumps delivering 1.4 mg/kg per hour (dose 1, *n* = 4), 2.5 mg/kg per hour (dose 2, *n* = 4), or 4.1 mg/kg per hour (dose 3, *n* = 4). Average 24-hour values for each group are given in the figure; *P < 0.05, **P < 0.01 versus Control. (b) Heart rate increased for Control mice and metoprolol treated mice receiving dose 1 and dose 2 (1.4 mg/kg per hour and 2.5 mg/kg per hour, resp.) during air-jet stress compared to baseline. For mice receiving metoprolol dose 3 (4.1 mg/kg per hour) heart rate did not increase during air-jet stress, compared to baseline. *P < 0.05 versus baseline. Mice received metoprolol treatment for one week prior to heart rate measurements. Data are expressed as mean ± SEM.

To find an appropriate dose of metoprolol for the atherosclerosis study we tested three different doses and their effects on heart rate. All three doses of metoprolol lowered resting heart rate during a 24-hour period. Although both doses 1 and 2 significantly reduced heart rate (Dunnett's, *P* < 0.05 and *P* < 0.01, resp.), the circadian rhythm was similar to the control situation. Dose 3, on the other hand, disrupted the circadian rhythm as well as markedly reducing heart rate (*P* < 0.01, Figure 1(a)). To further validate the metoprolol doses we performed air-jet stress. Air-jet stress markedly increased heart rate in control animals. Metoprolol doses 1 and 2 decreased the air-jet induced rise in heart rate; however, there was still a significant increase in heart rate. Only dose 3 abolished the heart rate response to air-jet stress (*P* < 0.05, Figure 1(b)). Hence, dose 2 was chosen for the following atherosclerosis study.

#### 3.1.2. Plasma Metoprolol Concentrations

Plasma concentrations of metoprolol were 79 ± 11, 119 ± 15, and 225 ± 18 nM, respectively, for the three different doses of metoprolol.

### 3.2. Study II: Effects on Atherosclerosis

#### 3.2.1. Metoprolol Reduced Atherosclerotic Plaque Area

**Figure 2:**
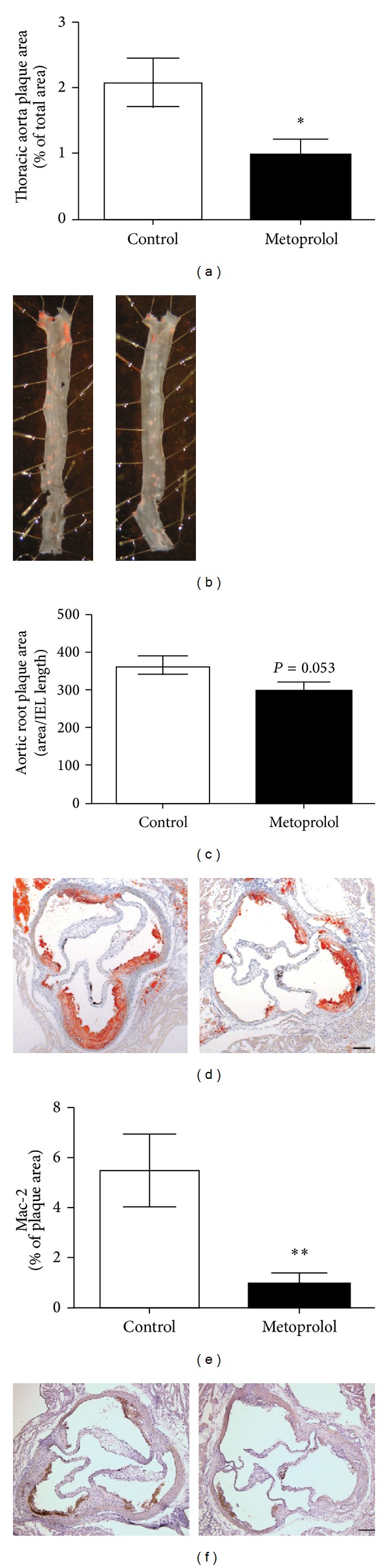
Metoprolol decreases atherosclerosis. (a)-(b) Metoprolol treatment (2.5 mg/kg per hour) decreased atherosclerotic plaque area in thoracic aorta. (c)-(d) A similar trend was seen in the aortic root, although this did not reach significance (*P* = 0.053). (e)-(f) Metoprolol treatment decreased the macrophage content in aortic lesions. Representative micrographs of thoracic aorta stained with Sudan IV (b), aortic root stained with Oil Red O (d), and macrophage marker Mac-2 (f). Left panel: Controls; right panel: metoprolol treated. Scale bar represents 200 *μ*m. *P < 0.05, **P < 0.01 versus Control.

Eleven weeks of metoprolol treatment significantly decreased atherosclerotic plaque area in thoracic aorta (*P* < 0.05, Figures 2(a) and 2(b)). A similar pattern was seen in the aortic root; however, this did not reach significance (*P* = 0.053, Figures 2(c) and 2(d)).

#### 3.2.2. Metoprolol Reduced Lesion Macrophages and Serum Levels of TNF*α* and CXCL1

Macrophage marker Mac-2 was significantly decreased in metoprolol treated compared to Control mice (*P* < 0.01, Figures 2(e) and 2(f)). Further, metoprolol treatment reduced serum levels of the Th1 cytokines TNF*α* and CXCL1, by approximately 30%, compared to untreated controls (*P* < 0.01, Table 1).

#### 3.2.3. Total Cholesterol

Metoprolol treatment was not associated with changes in total plasma cholesterol levels (Control 12.3 ± 0.5 versus Metoprolol 11.6 ± 0.4 mmol/L).

## 4. Discussion

In the present study we demonstrated that treatment with a moderate dose of the selective *β*_1_-adrenoceptor antagonist metoprolol reduced atherosclerotic plaque area in ApoE^−/−^ mice. Further, metoprolol reduced macrophage content in atherosclerotic lesions and decreased serum levels of the proinflammatory Th1 cytokines TNF*α* and CXCL1. Our study confirms the atheroprotective effect of metoprolol as previously reported [18] and complements the previous study by showing an effect on proinflammatory cytokines.

Different mechanisms may contribute to the decreased atherogenesis in the current study. Metoprolol is known to lower heart rate and reduce blood pressure. Hypertension is a well-known risk factor for atherosclerosis in humans and we cannot exclude that the antihypertensive effects of metoprolol explain the reduced atherogenesis in the present study. However, in normotensive patients metoprolol has little impact on blood pressure [11] and in normotensive mice blood pressure lowering seems to have little influence on atherogenesis [19]. The only valid way to study possible metoprolol effects on arterial blood pressure in the present experiments would be to perform long-term blood pressure telemetry experiments in ApoE^−/−^ mice with simultaneous chronic metoprolol infusions. We do not have such data. However, in a recent study investigating the effects of different *β*-antagonists on atherosclerosis none of the *β*-antagonists used, including metoprolol given at a similar dose as in the current study, influenced blood pressure [18]. Our suggestions are that blood pressure reduction may come into play but it is unlikely to be the sole effect on the decreased atherosclerosis.

Another, perhaps neglected, mechanism is the reduction in central sympathetic drive [20, 21]. The lipophilic property of metoprolol allows this *β*-blocker to enter the central nervous system and acts by decreasing sympathetic activity and increases vagal activation [22, 23]. This is of particular interest since recent studies show an anti-inflammatory signaling mediated via the vagus nerve [24]. Stimulation of the vagus nerve decreases release of the proinflammatory cytokine TNF*α* in a model of endotoxemia and vagotomy increases serum levels of TNF*α* [25]. It is interesting to speculate whether the anti-inflammatory effect of metoprolol could be attributed to decreased sympathetic activity and increased vagal tone. Indeed, in the current study we found decreased serum levels of the proinflammatory cytokines TNF*α* and CXCL1.

Previous studies show that metoprolol reduces the expression of adhesion molecules VCAM-1, ICAM-1, and MMP-1 in atherosclerotic lesions in rabbit aorta [26]. In line with this, we show that metoprolol reduces serum levels of TNF*α*, which is known to induce VCAM, ICAM, and MMPs [27]. Inhibition of TNF*α* or depletion of the TNF*α* gene reduces the progression of atherosclerosis in ApoE^−/−^ mice [28–30]. Transcription of TNF*α* is regulated by transcription factors such as NF-*κ*B [31], which has been shown to be activated by the sympathetic nervous system and to be inhibited by adrenergic blockade [32]. Hence, it is possible that metoprolol inhibits TNF*α* production through the inhibition of NF-*κ*B, with a subsequent reduction in expression of adhesion molecules.

The chemokine CXCL1, also known as KC (keratinocyte-derived chemokine), is the murine homologue of human GRO*α* (growth-regulated-oncogene-*α*). We have previously shown that sympathetic activation by social disruption stress in ApoE^−/−^ mice not only accelerates atherosclerosis but also increases plasma levels of CXCL1 [16]. On this note, we here demonstrate reduced serum levels by metoprolol treatment. CXCL1 and its receptor CXCR2 are present in atherosclerotic lesions and play an important role in adhesion of monocytes and accumulation of macrophages in lesions in mice [33–35]. Consequently, we found a decreased amount of macrophages in the atherosclerotic lesions after metoprolol treatment. Interestingly, beta receptor antagonism can also decrease the LDL affinity for arterial proteoglycans [36], thereby decreasing the accumulation of LDL in the arterial wall. In contrast, beta receptor antagonists may also decrease HDL levels and increase triglyceride levels, two proatherogenic features [36]. In the current study only total cholesterol levels were measured and we cannot exclude a change in lipoproteins. Nevertheless, it is plausible that not only decreased infiltration of macrophages but also reduced LDL accumulation could explain the decreased atherogenesis.

In this study we used a moderate dose of metoprolol, 2.5 mg/kg per hour. We chose this dose since it did not blunt heart rate reactivity during air-jet stress, allowing the mouse to react normally to a stressful situation. Further, heart rate was significantly decreased at baseline conditions during a 24-hour period, but more importantly the circadian rhythm was still virtually intact.

## 5. Conclusions

To conclude, metoprolol treatment reduces the progression of atherosclerosis in ApoE^−/−^ mice. A possible mechanism for the decreased atherogenesis is the anti-inflammatory effect of metoprolol, manifested as decreased serum levels of proinflammatory cytokines TNF*α* and CXCL1 and, consequently, decreased macrophage content in the atherosclerotic plaques.

## Figures and Tables

**Table 1:** Effects of metoprolol treatment on Th1/Th2 cytokines (Study II).

	Th1						Th2		
	IL-1*β* (pg/mL)	IL-2 (pg/mL)	IL-12 total (pg/mL)	IFN-*γ* (pg/mL)	TNF*α* (pg/mL)	CXCL1 (pg/mL)	IL-4 (pg/mL)	IL-5 (pg/mL)	IL-10 (pg/mL)
Control	4.8 ± 0.2	7.8 ± 0.5	3016 ± 106	4.0 ± 1.0	1.6 ± 0.1	241 ± 18	7.8 ± 1.7	7.2 ± 0.8	81.8 ± 14
Metoprolol	4.9 ± 0.5	21.3 ± 14	2904 ± 95	5.9 ± 3.3	1.1 ± 0.1	161 ± 18	13.0 ± 7.4	8.0 ± 1.7	121 ± 52

*P* value (Mann-Whitney)	0.712	0.538	0.389	0.735	**0.008**	**0.005**	0.601	0.951	1.000

Serum cytokine concentrations (pg/mL) were analyzed with the Mann-Whitney *U* test. Data are expressed as mean ± SEM. *P* < 0.01 was considered statistically significant.

## References

[1] Wikstrand J, Kendall M (1992). The role of beta receptor blockade in preventing sudden death. *European Heart Journal*.

[2] (1999). Effect of metoprolol CR/XL in chronic heart failure: metoprolol CR/XL Randomised Intervention Trial in Congestive Heart Failure (MERIT-HF). *The Lancet*.

[3] Ablad B, Bjorkman J-A, Gustafsson D, Hansson G, Ostlund-Lindqvist A-M, Pettersson K (1988). The role of sympathetic activity in atherogenesis: effects of *β*-blockade. *American Heart Journal*.

[4] Wikstrand J, Berglund G, Hedblad B, Hulthe J (2003). Antiatherosclerotic effects of *β*-blockers. *The American Journal of Cardiology*.

[5] Kaplan JR, Manuck SB, Clarkson TB, Lusso FM, Taub DM (1982). Social status, environment, and atherosclerosis in cynomolgus monkeys. *Arteriosclerosis*.

[6] Kaplan JR, Manuck SB, Clarkson TB, Lusso FM, Taub DM, Miller EW (1983). Social stress and atherosclerosis in normocholesterolemic monkeys. *Science*.

[7] Kaplan JR, Manuck SB, Adams MR, Weingand KW, Clarkson TB (1987). Inhibition of coronary atherosclerosis by propranolol in behaviorally predisposed monkeys fed an atherogenic diet. *Circulation*.

[8] Skantze HB, Kaplan J, Pettersson K (1998). Psychosocial stress causes endothelial injury in cynomolgus monkeys via *β*1-adrenoceptor activation. *Atherosclerosis*.

[9] Ostlund-Lindqvist AM, Lindqvist P, Brautigam J, Olsson G, Bondjers G, Nordborg C (1988). Effect of metoprolol on diet-induced atherosclerosis in rabbits. *Arteriosclerosis*.

[10] Hedblad B, Wikstrand J, Janzon L, Wedel H, Berglund G (2001). Low-dose metoprolol CR/XL and fluvastatin slow progression of carotid intima-media thickness: main results from the *β*-blocker cholesterol-lowering asymptomatic plaque study (BCAPS). *Circulation*.

[11] Wiklund O, Hulthe J, Wikstrand J, Schmidt C, Olofsson S, Bondjers G (2002). Effect of controlled release/extended release metoprolol on carotid intima-media thickness in patients with hypercholesterolemia: a 3-year randomized study. *Stroke*.

[12] Östling G, Gonçalves I, Wikstrand J, Berglund G, Nilsson J, Hedblad B (2011). Long-term treatment with low-dose metoprolol CR/XL is associated with increased plaque echogenicity: the Beta-blocker Cholesterol-lowering Asymptomatic Plaque Study (BCAPS). *Atherosclerosis*.

[13] Bernberg E, Andersson IJ, Tidstrand S, Johansson ME, Bergström G (2009). Repeated exposure to stressors do not accelerate atherosclerosis in ApoE^−/−^ mice. *Atherosclerosis*.

[14] Johansson ME, Bernberg E, Andersson IJ (2009). High-salt diet combined with elevated angiotensin II accelerates atherosclerosis in apolipoprotein E-deficient mice. *Journal of Hypertension*.

[15] Johansson ME, Wickman A, Fitzgerald SM, Gan L, Bergström G (2005). Angiotensin II, type 2 receptor is not involved in the angiotensin II-mediated pro-atherogenic process in ApoE^−/−^ mice. *Journal of Hypertension*.

[16] Bernberg E, Ulleryd MA, Johansson ME, Bergström GML (2012). Social disruption stress increases IL-6 levels and accelerates atherosclerosis in ApoE^−/−^ mice. *Atherosclerosis*.

[17] Johansson ME, Wickman A, Skøtt O, Gan L, Bergström G (2006). Blood pressure is the major driving force for plaque formation in aortic-constricted ApoE^−/−^ mice. *Journal of Hypertension*.

[18] Shimada K, Hirano E, Kimura T, Fujita M, Kishimoto C (2012). Carvedilol reduces the severity of atherosclerosis in apolipoprotein E-deficient mice via reducing superoxide production. *Experimental Biology and Medicine*.

[19] Lu H, Cassis LA, Daugherty A (2007). Atherosclerosis and arterial blood pressure in mice. *Current Drug Targets*.

[20] Gourine A, Bondar SI, Spyer KM, Gourine AV (2008). Beneficial effect of the central nervous system *β*-adrenoceptor blockade on the failing heart. *Circulation Research*.

[21] Parker GW, Michael LH, Hartley CJ, Skinner JE, Entman ML (1990). Central *β*-adrenergic mechanisms may modulate ischemic ventricular fibrillation in pigs. *Circulation Research*.

[22] Åblad B, Bjurö T, Björkman J-A (2010). Metoprolol, but not atenolol, reduces stress induced neuropeptide Y release in pigs. *Scandinavian Cardiovascular Journal*.

[23] Gullestad L, Pernow J, Bjurö T (2012). Differential effects of metoprolol and atenolol to neuropeptide y blockade in coronary artery disease. *Scandinavian Cardiovascular Journal*.

[24] Andersson U, Tracey KJ (2012). Neural reflexes in inflammation and immunity. *Journal of Experimental Medicine*.

[25] Borovikova LV, Ivanova S, Zhang M (2000). Vagus nerve stimulation attenuates the systemic inflammatory response to endotoxin. *Nature*.

[26] Liang C, Xiaonan L, Xiaojun C (2009). Effect of metoprolol on vulnerable plaque in rabbits by changing shear stress around plaque and reducing inflammation. *European Journal of Pharmacology*.

[27] Zhang H, Park Y, Wu J (2009). Role of TNF-*α* in vascular dysfunction. *Clinical Science*.

[28] Brånén L, Hovgaard L, Nitulescu M, Bengtsson E, Nilsson J, Jovinge S (2004). Inhibition of tumor necrosis factor-*α* reduces atherosclerosis in apolipoprotein E knockout mice. *Arteriosclerosis, Thrombosis, and Vascular Biology*.

[29] Ohta H, Wada H, Niwa T (2005). Disruption of tumor necrosis factor-*α* gene diminishes the development of atherosclerosis in ApoE-deficient mice. *Atherosclerosis*.

[30] Canault M, Peiretti F, Poggi M (2008). Progression of atherosclerosis in ApoE-deficient mice that express distinct molecular forms of TNF-alpha. *Journal of Pathology*.

[31] De Martin R, Hoeth M, Hofer-Warbinek R, Schmid JA (2000). The transcription factor NF-kappa B and the regulation of vascular cell function. *Arteriosclerosis, Thrombosis, and Vascular Biology*.

[32] Bierhaus A, Wolf J, Andrassy M (2003). A mechanism converting psychosocial stress into mononuclear cell activation. *Proceedings of the National Academy of Sciences of the United States of America*.

[33] Boisvert WA, Santiago R, Curtiss LK, Terkeltaub RA (1998). A leukocyte homologue of the IL-8 receptor CXCR-2 mediates the accumulation of macrophages in atherosclerotic lesions of LDL receptor-deficient mice. *Journal of Clinical Investigation*.

[34] Boisvert WA, Rose DM, Johnson KA (2006). Up-regulated expression of the CXCR2 ligand KC/GRO-*α* in atherosclerotic lesions plays a central role in macrophage accumulation and lesion progression. *The American Journal of Pathology*.

[35] Huo Y, Weber C, Forlow SB (2001). The chemokine KC, but not monocyte chemoattractant protein-1, triggers monocyte arrest on early atherosclerotic endothelium. *The Journal of Clinical Investigation*.

[36] Linden T, Camejo G, Wiklund O, Warnold I, Olofsson SO, Bondjers G (1990). Effect of short-term beta blockade on serum lipid levels and on the interaction of LDL with human arterial proteoglycans. *Journal of Clinical Pharmacology*.

